# Predicting Ion Channels Genes and Their Types With Machine Learning Techniques

**DOI:** 10.3389/fgene.2019.00399

**Published:** 2019-05-03

**Authors:** Ke Han, Miao Wang, Lei Zhang, Ying Wang, Mian Guo, Ming Zhao, Qian Zhao, Yu Zhang, Nianyin Zeng, Chunyu Wang

**Affiliations:** ^1^School of Computer and Information Engineering, Harbin University of Commerce, Harbin, China; ^2^Heilongjiang Provincial Key Laboratory of Electronic Commerce and Information Processing, Harbin University of Commerce, Harbin, China; ^3^Life Sciences and Environmental Sciences Development Center, Harbin University of Commerce, Harbin, China; ^4^Department of Neurosurgery, The Second Affiliated Hospital of Harbin Medical University, Harbin, China; ^5^Department of Instrumental and Electrical Engineering, Xiamen University, Xiamen, China; ^6^School of Computer Science and Technology, Harbin Institute of Technology, Harbin, China

**Keywords:** ion channel, machine learning, random forest, SVM, feature selection

## Abstract

**Motivation:** The number of ion channels is increasing rapidly. As many of them are associated with diseases, they are the targets of more than 700 drugs. The discovery of new ion channels is facilitated by computational methods that predict ion channels and their types from protein sequences.

**Methods:** We used the SVMProt and the k-skip-n-gram methods to extract the feature vectors of ion channels, and obtained 188- and 400-dimensional features, respectively. The 188- and 400-dimensional features were combined to obtain 588-dimensional features. We then employed the maximum-relevance-maximum-distance method to reduce the dimensions of the 588-dimensional features. Finally, the support vector machine and random forest methods were used to build the prediction models to evaluate the classification effect.

**Results:** Different methods were employed to extract various feature vectors, and after effective dimensionality reduction, different classifiers were used to classify the ion channels. We extracted the ion channel data from the Universal Protein Resource (UniProt, http://www.uniprot.org/) and Ligand-Gated Ion Channel databases (http://www.ebi.ac.uk/compneur-srv/LGICdb/LGICdb.php), and then verified the performance of the classifiers after screening. The findings of this study could inform the research and development of drugs.

## Introduction

Ion channels are the pathways for the passive transport of various inorganic ions across a membrane. The structure and function of cellular ion channels are the basis of life-sustaining processes, and their genetic variation, and dysfunction are related to the occurrence and development of many diseases (Gabashvili et al., 2007; Bagal et al., 2013; Cheng et al., 2018a,c). Usually, ion channels are in a closed state. Under particular stimuli, the channel protein conformation changes, and the probability of the ion channels opening increases. Based on their type of gate, ion channels are typically categorized into voltage-gated ion channels and ligand-gated ion channels (Wang et al., 2017a). On the binding of a ligand, a ligand-gated channel undergoes a conformational change that causes opening of the channel gate and ion flux. Voltage-gated ion channels predominantly contain potassium (K^+^), sodium (Na^+^), calcium (Ca^2+^), and anion channels (Shu-An et al., 2011). They are usually surrounded by four transmembrane segments of the same subunit. In these channels, there are some charged groups (potential sensors) that control the gate. When the membrane potential changes, the electric sensors undergo a displacement under the effect of the electric field force, and the gate is opened or closed in response to the change in the membrane potential. Ion channels are expressed in practically all tissues and can cause deafness, renal cysts, cardiac arrhythmias migraines, and epilepsy (Cai et al., 2002a). Therefore, many drugs are found to target ion channels. One example is an antiarrhythmic drug, Lidocaine, which acts as a voltage-gated sodium channel inhibitor (Peters et al., 1993; Tiwari and Srivastava, 2015). The actions of Lidocaine affect the conduction system and muscle cells of the heart, raising its depolarization threshold and making it less likely to initiate or conduct action potentials (Lin et al., 2015). Another example is Ziconotide, which targets calcium channels and is used for pain relief. This compound blocks the calcium influx in the nerve terminals, which results in a reduced release of glutamate and neuropeptides, effectively interrupting the spinal transmission of pain signals (Schmidtko et al., 2010).

Owing to the significance of ion channels in biological processes, researchers have initiated conducting more in-depth research on them to establish the relationships between ion channels and different diseases. Currently, ion channels have become important targets for disease diagnosis and drug development. It is known that many chemicals and genetic disorders can disrupt the normal function of ion channels and have catastrophic consequences for living organisms (Santos et al., 2017). Most animal toxins are used to treat diseases such as chronic pain by modulating ion channels to shut down the nervous system.

In recent years, ion channels have played an increasingly important role in the treatment of diseases and drug research and development. Therefore, several researchers have started to pay attention to the structure and function of ion channels. With the rapid growth of proteomics data, earlier prediction and identification of the type of a particular ion channel has become important. Therefore, researchers have developed various bioinformatics software to predict the identification of ion channels. As researchers are interested in developing drugs that target ion channel and extending ion channel protein annotation, a series of high-throughput computational tools have been developed to predict ion channels and their types directly from protein sequences. In the last decade, many computational methods have been developed based on machine learning algorithms (Yu et al., 2015; Zou et al., 2017a,b; Stephenson et al., 2019), which are used in different fields, such as drug repositioning (Yu et al., 2016, 2017). Increasingly, researchers have applied machine learning algorithms to predict and classify ion channels. Sudipto et al. (2006) used amino acid composition and dipeptide composition as the feature vectors and classified them using a support vector machine (SVM) to predict voltage-gated ion channels and their subtypes. Liu et al. (2010) proposed a voltage-gated potassium channel identification method based on local sequence information. The prediction result of this method was better than that of voltage-gated potassium channel identification based on global sequence information (Lin and Ding, 2011). Zhao et al. (2017) constructed a support vector machine (SVM)-based model to quickly predict ion channels and their types. By considering the residue sequence information and their physicochemical properties, a novel feature-extracted method which combined dipeptide composition with the physicochemical correlation between two residues was employed. Recently, Gao et al. (2016) proposed a model based on a SVM to search for predicted ion channels and their subfamilies using the sequence similarity search feature of the basic local alignment search tool. Although many classifiers have been developed for the identification of ion channels, there are still some unresolved problems. For example, ion channel sequence similarity is very high, which may result in overestimation of the predictive classification performance of the model (Olivier and Du, 2012).

In this study, SVM and random forest classifiers were used to identify ion channels and further classify them. The maximum-relevance-maximum-distance (MRMD) method was introduced for feature selection to improve the prediction accuracy. We followed three steps to predict and classify ion channels. First, a protein sequence was detected to determine if it belonged to an ion channel. If the test results demonstrated that the sequence was an ion channel, then the protein sequence was classified as either a voltage-gated ion channel or ligand-gated ion channel. Finally, if the protein sequence was found to belong to a voltage-gated ion channel, we classified it as a potassium (K^+^), sodium (Na^+^), calcium (Ca^2+^), or anion voltage-gated ion channel.

## Materials and Methods

**Figure 1:**
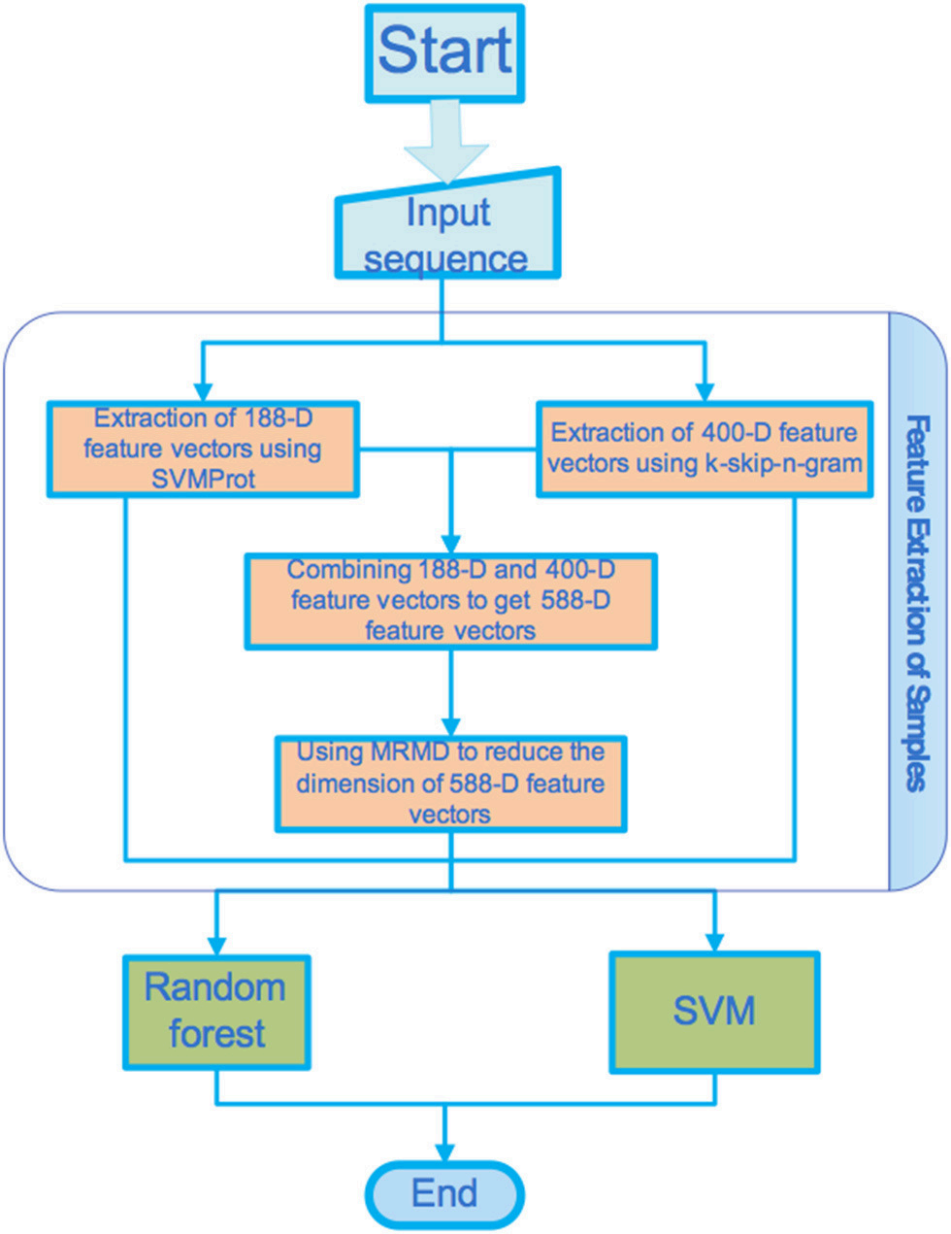
Workflow of the proposed processes.

Figure 1 shows the basic flow of the processes proposed in this paper. In this section, we introduce in detail the data set, feature extraction method, dimension reduction method, and classifier used in this study.

### Benchmark Dataset

The data that we used to establish the prediction model in this study were collected from Lin and Ding (2011). The sequences of ion channels were collected from the Universal Protein Resource (UniProt) and Ligand-Gated Ion channel databases (Marco et al., 2006). The following measures were taken to obtain reliable high-quality datasets. Initially, the protein sequences containing blurred disabilities, such as those with amino acids “X,” “B,” and “Z” were discarded. Then, the sequences of other protein fragments were removed. Proteins that were inferred by homology or prediction were discarded because of their unreliability. Finally, to avoid any homology bias, the CD-HIT (Li and Godzik, 2006) program was used to remove highly homologous sequences, with a 40% sequence identity as the cutoff (Wei et al., 2012; Chen et al., 2016; Zou et al., 2018a).

In strict accordance with the above steps, 148 voltage-gated ion channels, including 81 potassium channels, 29 calcium channels, 12 sodium channels, 26 anion channels, and 150 ligand-gated ion channels were finally extracted. To ensure the reliability and practicability of the ion channel prediction, and classification and maintenance of the balance between the positive and negative data, 300 protein sequences were randomly selected from UniProt as non-ion channels. It was observed that the consistency of these non-ion channel sequences was < 40%.

### Feature Extraction of Samples

Section Benchmark dataset mainly discusses the series of preprocessing steps performed for the dataset. The reconstruction provided a reliable database for the study on the positioning method. This section focuses on specific methods of protein subcellular localization based on machine learning.

The first and most important role of a predictor is to extract protein sequences (Liu et al., 2015; Ding et al., 2017a,b; Zou et al., 2018b). We used two feature extraction methods including the SVMProt 188-D feature extraction method, which is based on protein composition and physicochemical properties, and the k-skip-n-gram 400-D feature extraction method.

#### SVMProt 188-D Feature Extraction

Different types of amino acids possess their own unique physicochemical properties. These characteristics of amino acid sequences can be used to predict types of protein. This method has yielded good predictive results (Cao and Cheng, 2016; Li et al., 2016b). Dubchak et al. (1995) proposed a composition transition distribution model based on the composition, transformation, and distribution of protein sequences, and achieved better results for the prediction of protein folding patterns. The physicochemical properties of protein sequences were fully embodied in this model, where the composition and physical and chemical properties were independent of each other. Cai et al. (2003) extracted 188-dimensional features in combination with amino acid composition and physicochemical characteristics for the characterization of proteins. SVMProt also contains nine physicochemical properties besides amino acid frequencies. The quantities of each of these properties are listed in Table 1 (Zou et al., 2013a,b).

**Table 1:** Number of features in SVMPRot.

**Ordinal**	**Physicochemical characteristics**	**Dimension**
1	Amino acids composition	20
2	Hydrophobicity	21
3	Normalized van der Waals volume	21
4	Polarity	21
5	Polarizability	21
6	Charge	21
7	Surface tension	21
8	Secondary structure	21
9	Solvent accessibility	21

In the model, 20 amino acids in the query protein sequence constitute the first 20-dimensional feature vector. The first 20-dimensional vector is calculated as follows:

(1) $$E_{i}=\frac{A_{i}}{L}\times 100\%\left(1\leq i\leq 20\right)$$

where A_i_ and L denote the number of the amino acids in the sequence and the length of the sequence, respectively, (Zhu et al., 2018b A20). {A_1_, A_2_, …, A_20_} represents the 20 amino acids that form the proteins. According to the physicochemical types, the amino acids can be classified under three categories based on their content (C), distribution (D), and bivalent frequency (F) (Bagal et al., 2013). The features of each of the remaining eight physicochemical properties are obtained using the following formula:

(2) $$C_{i}=\frac{count_{D_{i}}}{L}\times 100\left(1\leq i\leq 20\right)$$

$$T_{i,j}=\frac{D_{i}D_{j}\;orD_{j}D_{i}}{L-1}\times 100,$$

(3) i,j∈{(i=c,j=d),(i=c,j=f),(i=d,j=f)}

$$D=\frac{P_{j}th\;ofD_{i}}{L}\times 100,$$

(4) (j=0,1,2,3,4;i=c,d,f)

and

(5) $$P_{j}=\{\begin{array}{c} 1\\ \frac{count_{D_{i}}}{4\times j} \end{array}\;\left(j=1,2,3,4\right)$$

where D_i_ (i = c, d, f) and *count*_*D*_*i*__denote the physicochemical properties of the amino acids and number of such properties present in the sequence, respectively. After calculating all the physical and chemical properties, we finally extracted all the 188 (20 + (21 × 8) = 188) feature vectors.

#### k-skip-n-gram 400-D Feature Extraction

Guthrie et al. (2006) first proposed the k-skip-n-gram model. In protein sequences, the distance between two amino acids Ai and A_j_ is denoted by DT (A_i_, A_j_), which is defined as the position interval between two amino acids (Liu et al., 2014). It is calculated as follows:

(6) $$DT\left(A_{i},A_{j}\right)=j-i-1$$

where i and j are the positions of the amino acids in a sequence.

The k-skip-n-gram model provides the composition of n residues with distances k in a sequence. Its features are calculated as follows:

$$FV_{SkipGram}=\{\frac{N\left(a_{m_{1}}a_{m_{2}}\ldots a_{m_{n}}\right)}{N\left(T_{SkipGram}\right)}|$$

(7) $$1\leq a_{m_{1}}\leq 20,1\leq a_{m_{2}}\leq 20,\ldots ,1\leq a_{m_{n}}\leq 20$$

where *N*(*T*_*SkipGram*_)and *N*(*a*_*m*_1__*a*_*m*_2__…*a*_*m*_*n*__)denote the total number of elements in set *T*_*SkipGram*_ and total number of terms *a*_*m*_1__*a*_*m*_2__…*a*_*m*_*n*__ appearing in set *T*_*SkipGram*_, which is formulated as

(8) $$T_{SkipGram}=\left\{\cup_{a=1}^{k}Skip\left(DT=a\right)\right\}$$

where

(9) $$\begin{array}{l} \;Skip\left(DT=a\right)\\ =\left\{A_{i}A_{i+a+1}\ldots A_{i+a+n-1}|1\leq i\leq L-a,\;1\leq a\leq k\right\} \end{array}$$

Because only 20 amino acids can form a protein, a sequence has a total of 20^n^ permutations. Therefore, a protein sequence can be transformed into 20^n^ feature vector sets *FV*_*SkipGram*_.

As the number of feature vectors exhibits an exponential distribution, the value of n is quite important. When *n* = 1, there are only 20 features. If the number of features is quite small, the feature representation of a sequence is negatively affected. In contrast, when the value of n is very high, it affects the calculation efficiency. In this study, the value of n was considered as 2. Finally, we obtained 400 feature vectors.

### Feature Selection (MRMD)

Owing to their limitations, the two feature representation methods mentioned above were combined to form a new feature vector containing more than one feature. SVM and random forest classifiers were used to classify the new feature vector set. When multiple feature extraction methods are combined, many dimensions may be generated and the classification result may be affected (Tang et al., 2017; Liu et al., 2018b; Zhu et al., 2018b). Feature selection can alleviate the problem of dimensionality by selecting a subset of features (Zhu et al., 2018c). Therefore, we employed the dimensionality reduction method based on MRMD (http://lab.malab.cn/soft/MRMD/index_en.html) to reduce the dimensionality of the generated feature vectors (Xu et al., 2016; Zou et al., 2016a,b; Zhu et al., 2017, 2018b; Chen et al., 2018; Tang et al., 2018b). MRMD selects the feature with the highest correlation and least redundancy by calculating the maximum relevance and maximum distance. In this study, Pearson's correlation coefficients were used to measure the relevance, and three distance functions were used to calculate the redundancy of the features. As the value of the Pearson correlation coefficient increased, the relationship between the features and target classes became stronger. As the distance between the features increased, the redundancy of the feature vectors decreased. Finally, the sub-features generated after the MRMD dimension reduction were found to possess the characteristics of low redundancy and a strong relationship. This could aid in achieving more accurate classification results.

### Classifier Models

#### Random Forest

A random forest is a classifier that uses multiple trees to train and predict samples; it has been widely used in many bioinformatics tasks (Xu et al., 2013, 2018b; Liu et al., 2018a; Pan et al., 2018; Su et al., 2018; Wei et al., 2018a). It was proposed by Leo Breiman in 2001 and combines the Bagging integrated learning theory with the random subspace method (Verikas et al., 2011). A random forest is an integrated learning model based on a decision tree. It contains multiple decision trees trained by the Bagging integrated learning technology. Samples are input into a random forest for classification. The final classification result is governed by the output of a single decision tree. Since Buntine and Niblett (1992) proposed the random forest algorithm, it has been widely used, owing to its good performance, in many practical fields, such as the classification and regression of gene sequences, action recognition, face recognition, anomaly detection in data mining, and metric learning. In this study, we used a random forest classifier to build a model.

#### Support Vector Machine

**Figure 2:**
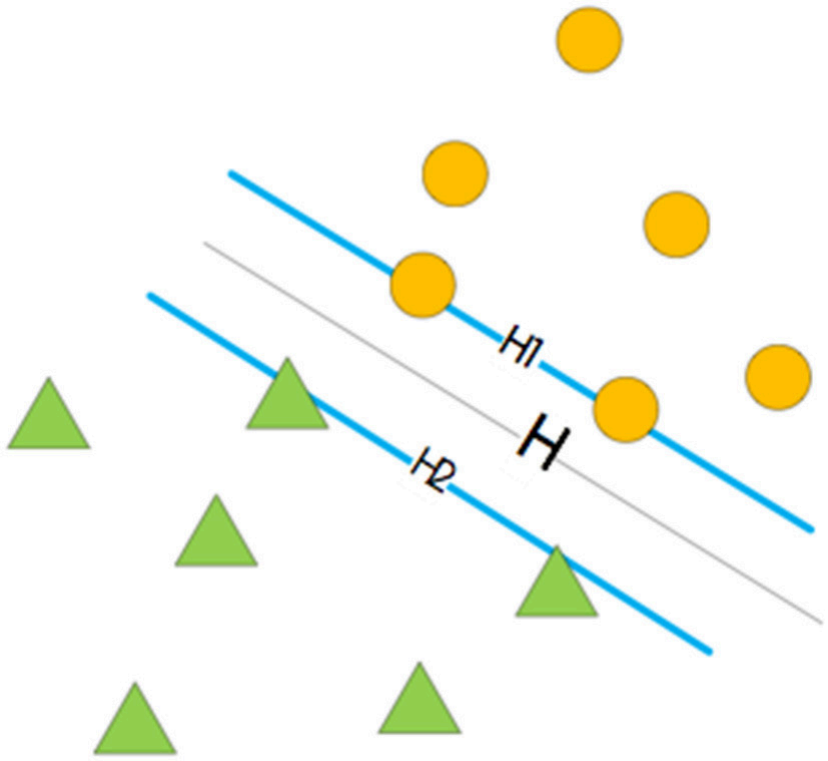
Optimal hyperplane of SVM.

An SVM is a supervised learning model related to learning algorithms and has achieved good performance in several bioinformatics (Momot et al., 2010; Cao et al., 2014; Ding et al., 2016; Li et al., 2016a; Wang et al., 2017b, 2018; Wei et al., 2017a,b, 2018c; Chen and Chuang, 2018; Liu et al., 2018c; Tang et al., 2018a; Shen et al., 2019; Zhu et al., 2019) and biomedicine (Zeng et al., 2018a; Zhang et al., 2018) studies. The dual-classification problem of an SVM can be broadly divided into three cases: linear separable, approximate linear separable, and non-linear separable. The solution for the linear separable problem is an optimal hyperplane that allows two groups of samples to be classified appropriately and to have the largest classification interval. This is shown in Figure 2, where the H plane is the optimal hyperplane. The approximate linear separability problem can be solved by adding a relaxation variable, i, in the optimization function of the linear classification. To solve the non-linear separable problem, we need to select an appropriate kernel function, transform the low-dimensional space into a high-dimensional space, and find the appropriate classification plane in the high-dimensional space so that the two samples can be classified appropriately (Cai et al., 2002b; Yu-Dong et al., 2010; Liu, 2017). Therefore, an SVM can achieve good classification results even when there are few experimental data. In this study, we used LIBSVM 3.23, which was downloaded from https://www.csie.ntu.edu.tw/~cjlin/libsvm/index.html. To obtain the optimal model, we performed a grid search to optimize parameters c and g. Then, the values of c and g were added to the model to obtain the optimal classification result. A combination of different types of features and classifiers can improve the overall performance of the model (Zhu et al., 2016, 2018a).

### Prediction Assessment

In machine learning, dividing experimental data into training sets is necessary to build a prediction model (Cao et al., 2017; Xu et al., 2017; Cheng et al., 2018b; Hu et al., 2018). Experimental data need to be further divided into test sets so that the final results of the training can be validated. To divide experimental data into training and test sets, a large amount of experimental data is needed. However, in practice, the number of experimental data is often limited. Therefore, researchers often use cross-validation for testing. Three types of cross-validation methods are commonly used in bioinformatics: independent data testing, folding cross-validation, and n-fold cross-validation. Among these, the folding knife test has been widely used in bioinformatics owing to its excellent results. However, this test is time and resource intensive (Lin et al., 2012; Zeng et al., 2016; Lai et al., 2017; Liu et al., 2017b; Manavalan et al., 2018). The n-fold cross-validation is commonly used to test the accuracy of an algorithm. The dataset was divided into 10 parts, nine of which were used as the training data and one as the testing data. After several experiments were conducted using numerous amounts of varied data, the best error estimates were obtained by dividing the dataset into 10 parts. There is sufficient theoretical basis to prove this approach (Chen et al., 2017; Zeng et al., 2018b).

### Performance Evaluation

To obtain clearer classification prediction results and estimate the accuracy of the prediction model, we used other evaluation criteria as well (Feng et al., 2013, 2018; Chen et al., 2017; Zhang and Liu, 2017; Dao et al., 2018; Yang et al., 2018). The prediction accuracy was estimated using the sensitivity (Sn), overall accuracy (OA), and average accuracy (AA), which are defined as follows:

(10) $$Sn\left(i\right)=\frac{TP_{i}}{TP_{i}+FN_{i}}$$

(11) $$OA=\sum_{i=1}^{n}\frac{TP_{i}}{N}$$

and

(12) $$AA=\sum_{i=1}^{n}Sn(i)/n$$

where TP_i_ and FN_i_ denote the true positives and false positives of the ith class, respectively, (Liu et al., 2017a; Zeng et al., 2017a). N and n are the total number of sequences and number of species, respectively.

## Results

### Prediction Results of Ion and Non-ion Channels

We compared the predictive effects of the SVM-based and random forest-based methods on both ion and non-ion channels in different dimensions. The results obtained are listed in Table 2. The 10-fold cross-validation results of the 188-dimensional features, 400-dimensional features, and mixed features (188-dimensional features combined with 400-dimensional features) are listed in Table 2. We then applied the MRMD method to reduce the dimensions of the 588-dimensional features to obtain 587-dimensional features. However, the average classification accuracy of the 587-dimensional features was found to be lower than that of the 400-dimensional features. The results also revealed that the SVM classifier was the best method for classifying the 400-dimensional features, with an average overall accuracy (OA) rate of 85.1%. As can be seen in Table 2, 86.6% of the ion channels and 83.7% of the non-ion channels can be appropriately identified using the SVM classifier, with a total accuracy rate of 85.1%. The feature vectors of the 188- and 400- dimensional features yield good prediction results. This result reveals that the SVM can moderately improve the predictive performance of the model. And we also try to use other classifiers to classify ion channels, but the classification effect is obviously worse than that of random forest and SVM classifiers, so we finally choose the two classifiers for comparison.

**Table 2:** Prediction results of ion channels and non-ion channels.

**Method**	**Ion channel (%)**	**Non-ion channel (%)**	**OA (%)**
Random forest (188D)	90.3	77.2	83.7793
SVM (188D)	87.0	78.5	82.7759
Random forest (400D)	87.7	77.5	82.6087
SVM (400D)	86.6	83.7	85.1171
Random forest (588D)	77.5	90	83.7793
SVM (588D)	83.2	80	81.6054
Random forest (587D)	77.2	89.7	83.4448
SVM (587D)	77.2	83.3	80.2676

### Classification Results of Voltage-Gated and Ligand-Gated Ion Channels

We evaluated the accuracy of the 188-dimensional features, 400-dimensional features, and mixed features (188-dimensional features combined with 400-dimensional features), and the 88-dimensional features obtained after the dimensional reduction using the MRMD method for discriminating between the classification results of voltage-gated and ligand-gated ion channels. The results are tabulated in Table 3. They reveal that the random forest classifier is the best for classifying the 188-dimensional features, with an average overall accuracy rate of 89.9%. As seen in Table 3, 93.9% of the voltage-gated ion channels and 86.0% of the ligand-gated ion channels could be correctly identified using the random forest method. The results reveal that the random forest classifier is better than the SVM classifier in some cases and can improve the prediction performance model.

**Table 3:** Prediction results of voltage-gated and ligand-gated ion channels.

**Method**	**Voltage-gated ion channels (%)**	**Ligand-gated ion channels (%)**	**OA (%)**
Random forest (188D)	93.9	86.0	89.9329
SVM (188D)	91.9	86.7	89.2617
Random forest (400D)	88.5	82.7	85.5705
SVM (400D)	82.4	83.3	82.8859
Random forest (588D)	89.2	86.0	87.5839
SVM (588D)	91.9	86.7	89.2617
Random forest (188D)	92.6	86.7	89.5973
SVM (188D)	91.9	86.7	89.2617

The results listed in Tables 2, 3 reveal that the difference between the voltage-gated and ligand-gated ion channels appears to be more distinct than that between the ion and non-ion channels. This may be due to the obvious differences between voltage-gated ion channels and ligand-gated ion channels with respect to some specific components.

### Classification Results of Four Types Voltage-Gated Ion Channels

Finally, we classified the four types of voltage-gated ion channels, i.e., K, Ca, Anion, and Na, using the SVM and random forest methods. The prediction accuracy of the 188-dimension features, 400-dimensional features, 424-dimensional features, and mixed features were calculated individually. The results are listed in Table 4. This table shows that the best classification effect is achieved when the SVM classifier, which had a maximum overall accuracy rate of 72.973%, is used to extract the 188-dimensional features. We applied the MRMD method to reduce the dimensions of the 588-dimensional features to obtain 424-dimensional features. However, the average classification accuracy of the 424-dimensional features was lower than that of the 188-dimensional features. After dimension reduction, the dimension of ion channel feature vectors did not decrease significantly, and the accuracy was even decreasing, which indicates that MRMD was not effective in classifying ion channel feature vectors.

**Table 4:** Prediction results for four types of voltage-gated ion channels.

**Method**	**K (%)**	**Ca (%)**	**Na (%)**	**Anion (%)**	**OA (%)**	**AA (%)**
Random forest (188D)	97.5	37.9	50	46.2	72.973	57.9
SVM (188D)	96.3	48.3	58.3	69.2	79.0541	68.0
Random forest (400D)	97.5	6.9	50	23.1	62.8378	44.4
SVM (400D)	85.2	62.1	50	73.1	75.6757	67.6
Random forest (588D)	97.5	34.5	50	57.7	74.3243	59.9
SVM (588D)	96.3	48.3	58.3	69.2	79.0541	60.2
Random forest (424D)	98.8	34.5	58.3	46.2	73.6486	59.5
SVM (424D)	96.3	48.3	58.3	69.2	79.0541	68.0

In general, the robustness of the results can be improved by using the minimum dimensions of the feature vector data. Therefore, we recommend using 188-dimensional feature vectors to predict the four types of voltage-gated ion channels.

## Discussion and Conclusions

In this study, new features were used to extract the features of ion channels, and good prediction results were obtained. To accurately predict and classify ion channels and their types, we constructed SVM-based and random forest-based models that used SVMProt 188- dimensional feature extraction and k-skip-n-gram to extract features. Then, we combined the 188-dimensional features with the 400-dimensional features to obtain 588-dimensional features. To achieve a higher accuracy with fewer features, the MRMD method was used to reduce the dimensions of the 588-dimensional features. Finally, the SVM and random forest models were used to model 188-dimensional features, 400-dimensional features, 588-dimensional features, and the MRMD-reduced features. The experimental results revealed that the features extracted by the SVMProt 188-dimensional feature extraction and k-skip-n-gram methods could effectively predict and classify the ion channels. Such a fast and accurate method can accelerate the prediction of ion channels and promote the discovery of drug targets.

Although this method can guide the study of ion channel discovery, it has some limitations. With the rapid increase in ion channel types and data, more perfect prediction and classification models need to be developed by researchers. We believe that more in-depth research using computational intelligence (Mrozek et al., 2009; Zeng et al., 2014; Cabarle et al., 2017; Xu et al., 2018a) and machine learning (Zeng et al., 2017b; Song et al., 2018; Zhu et al., 2018c) can result in the development of additional feature extraction methods (Wei et al., 2018b) and more accurate prediction classification models (Wang et al., 2016), and contribute to drug research and development.

## Data Availability

The raw data supporting the conclusions of this manuscript will be made available by the authors, without undue reservation, to any qualified researcher.

## Author Contributions

KH, MW, LZ, and YW made substantial contributions to the design of the work and drafted and revised the article. MG, MZ, QZ, and YZ focused on the machine learning programs and plotted the figures. NZ and CW mainly made the analysis and interpretation of data for the work.

### Conflict of Interest Statement

The authors declare that the research was conducted in the absence of any commercial or financial relationships that could be construed as a potential conflict of interest.
